# Engaged scholarship and public policy decision-making: a scoping review

**DOI:** 10.1186/s12961-020-00613-w

**Published:** 2020-08-26

**Authors:** Jessie-Lee D. McIsaac, Barbara L. Riley

**Affiliations:** 1 Faculty of Education and Department of Child and Youth Study, Mount Saint Vincent University, 166 Bedford Highway, Halifax, Nova Scotia B3M 2J6 Canada; 2 Healthy Populations Institute, Dalhousie University, Halifax, Nova Scotia B3H 4R2 Canada; 3 Faculty of Applied Health Sciences and Renison University College, University of Waterloo, 200 University Avenue West, Waterloo, Ontario N2L 3G1 Canada

**Keywords:** Engaged scholarship, research partnerships, collaboration, public policy

## Abstract

**Background:**

Engaged scholarship includes the coproduction and use of research by partnerships that blend research, policy and/or practice perspectives. This way of doing research attempts to bridge-the-gap between knowledge and its application. Recent reviews have described practices that support engagement and involve the community in research and patients in healthcare but there is less known about how to engage individuals working to inform public policy.

**Aims and objectives:**

The purpose of this research was to articulate the actions and context that support the coproduction and use of research to inform public policy decisions. The study focuses on partnerships between researchers and stakeholders working in public policy across different levels and sectors of government.

**Methods:**

A scoping review methodology was used. Relevant articles were identified from six electronic bibliographic databases of peer-reviewed literature.

**Findings:**

A total of 9904 articles were screened and 375 full-text articles were assessed for eligibility. The included 11 studies were from research partnerships internationally and described actions and contextual factors contributing to the coproduction and use of research to inform public policy. Key actions included facilitating frequent interactions with public policy stakeholders, joint planning for research, and collaboration to execute data collection and analysis. Contextual factors included clarity in responsibilities, prior relationships, and mutual respect for partner priorities and perspectives.

**Conclusions:**

Key actions and contextual factors were identified in this review and warrant further study to strengthen research–policy partnerships and their outcomes.

## Background

Engaged scholarship (ES) offers a collaborative approach to research where academicians and non-academicians (e.g. community, policy-makers and other partners) engage in the coproduction and use of knowledge. This way of doing research attempts to bridge the gap between knowledge and action by blending the perspectives of those who produce knowledge and those who use knowledge as part of the research process [1]. Forms of ES have emerged across university institutions to affirm the commitment of academia to the scholarship of engagement and address complex population and social issues [2–5]. The characterisation and experiences of ES have focused on research, instruction, service and commercialisation and academic experiences across disciplines of health research, social science, engineering, computer science, information technology and business [2, 4–7]. As a result of the diversity, ES experience and involvement is variable and shaped by discipline, language, institutional culture, individual roles and understandings [3].

Within the domain of ES for research, the focus is on discovery and inquiry in collaboration with a broad set of community partners that are affected by the issues (e.g. community members, patients, professionals, organisations) and/or by decision-makers (e.g. policy-makers, leaders, managers) who may apply research findings [4, 8]. With roots in social sciences, ES emphasises multidirectional learning where different expertise is valued and shared to inform better quality and more relevant research [9]. This is distinctly different from a traditional biomedical knowledge transfer paradigm, where researchers are responsible for doing research and communicating the results to end users [9]. Other terms have been used to describe more recent efforts to engage stakeholders in the co-development of research (e.g. integrated knowledge translation, knowledge mobilisation, dissemination and implementation) and each has its own paradigmatic influences and underpinnings [10]. Biomedical roots in health may limit the extent to which traditions from the social sciences and humanities, like ES, are used to understand and improve research partnerships.

Recent reviews involving ES have explored the roles and implications of community partners in research. These reviews demonstrate variability in practices that facilitate engagement and influence community mobilisation and empowerment as a result of the practice of ES, including trust, linkages, training, resources, institutional processes and sustainability [8, 11–15]. Studies have also highlighted factors influencing the use of research by policy decision-makers such as the perceptions of evidence, culture and competing influences, and practical constraints [16, 17]. In population health research, engaging stakeholders working in public policy in research is particularly important to ensure that evidence is produced in a timely manner to influence policy decisions [18, 19]. ES provides an opportunity to bring together explicit or codified knowledge that is typically represented in scientific literature, with tacit or experiential knowledge that is based on professional expertise and involvement with local communities [20], which can be important in the process of programme planning and decision-making [21]. However, little is known about how those working in public policy, such as government decision-makers and staff, should be engaged in research to bridge the research-to-policy gap. This includes conditions and actions of ES partnerships to optimise public policy and is the focus of the present study.

An extensive and diverse literature addresses questions about research–policy partnerships, particularly in relation to factors that facilitate the use or uptake of research knowledge [22, 23]. Yet, it is unclear what conditions and actions of ES partnerships best support the desired public policy outcomes [24]. Scoping reviews offer a review method to summarise the extent and range of literature on a given topic and to identify gaps in the literature [25]. The purpose of this research was to conduct a scoping review to articulate the actions and context that support the coproduction and use of research knowledge to inform public policy decisions. It was expected that this review would identify strategies for stakeholders working in ES partnerships to enhance their collaborative research efforts and ascertain key areas that require future investigation.

## Methods

The review was guided by the scoping review methodological framework by Arksey and O’Malley [25] and by further recommendations by Levac et al. [26]. The review followed five key steps, including (1) identifying the research question; (2) identifying relevant studies; (3) study selection; (4) charting the data; and (5) collating, summarising and reporting the results.

The contextual sensitivity of a realist lens was used to inform the research question to identify what ES actions work for whom, in what circumstances, in what way and how [27]. We used the word ‘action’ rather than ‘mechanism’, which is traditionally used in realist approaches as the review is not about theory-building related to underlying (invisible) mechanisms [28]. We adapted Denyer and Tranfield’s approach to developing a well-formulated question for use in a social science or organisational context, which is also informed by a realist approach and uses the CIAO acronym (context, intervention, actions and outcomes) [29]. Our final research questions were – Under what conditions does ES influence the coproduction and use of research to inform public policy? What are the actions that contribute to sustained ES partnerships between researchers and government stakeholders working in public policy? For the purpose of our review, we defined public policy as a government action or inaction that includes formally approved and implemented goals and regulations, practices and programmes [30]. This research question informed the inclusion and exclusion criteria, which were refined iteratively throughout the search process.

The research question informed search concepts to identify relevant studies, with searches performed using the terms ‘Policy Makers’ AND ‘Partnership’ AND ‘Research’. The team identified example articles within the target subject area. A search strategy was then developed for use in Ovid MEDLINE and tested for retrieval of the target articles. Once finalised, the search strategy was translated to five other databases (Ovid MEDLINE, Embase, Web of Science, ABI/INFORM Global, PAIS Index, ERIC). Additional file 1 provides a summary of the search translation for all databases, which includes the literature up until May 2017. Following de-duplication of identified articles, an iterative process was used for study screening. Two reviewers (KM/BB and JLM) independently reviewed titles and abstracts, followed by the full articles within the Covidence platform. Reviewers met to discuss discrepancies and refine the inclusion/exclusion criteria (Table 1) as needed [26]. For example, as studies were screened, it became clear that it would be important to focus the screening on collaborations that were enduring to ensure they reflected the intent of a ES partnership, rather than isolated meetings without a clear long-term collaborative purpose.

**Table 1:** Inclusion and exclusion criteria for scoping review

	Inclusion	Exclusion
**General criteria**	Publication time scan (2000–2017); English language; any study type and design (e.g. descriptive, experimental, qualitative, quantitative)	Publication time scan (before 2000); not English language
**Context**	Includes researchers and individuals working in public policy in any level of government (local/municipal, provincial/state, federal/national) across low-, middle- and high-income countries and descriptions of their environments (socio-cultural, political, economic); may also include other stakeholders (e.g. community, providers/practitioners, patients/public)	Does not involve researchers or individuals working in government (e.g. community, organisational, administrative or clinical); no description of environments
**Intervention** **(in this study meaning the engaged scholarship partnership)**	Description of experiences working together toward the same end goal; focused on collaborative coproduction and use of knowledge (e.g. involvement in teams, developing research/policy questions, designing and conducting methods, disseminating results); enduring collaboration that goes beyond one project/meeting	Researchers working independent of decision-makers and vice versa; only translating information to public policy decision-makers, focusing only on uptake of evidence or policy relevance; a network without an enduring mutual purpose; an isolated meeting or project without a long-term collaboration; focus on participatory policy-making rather than on using research to inform policy; focus on a network without information about the specific use of research to inform policy-making
**Actions**	Clear and concrete examples of processes/steps that outline what was done to engage and support collaborations	Not a direct account of an experience of engaged scholarship (no or vague details on the process of how it was implemented such as a commentary or descriptive paper on the topic of engaged scholarship)
**Outcomes**	Coproduced knowledge will inform public policy	Not informing public policy

Data from the final articles were charted to extract information according to a priori characteristics, including title, authors, year, journal, country, public policy issues, type of ES partnership described, methods used to describe or evaluate, stakeholders (research, public policy, other), initiator, funding, duration, actions (including duration, frequency, timing), contextual factors (barriers and facilitators), and public policy outcomes. From the extracted data, we used a thematic analysis approach [31] to code data from the included study and identify themes that related to the key conditions and actions to summarise and report the results.

## Results

A total of 9904 articles were screened (Table 2). Of these, 375 full-text articles were assessed for eligibility (see Additional file 2 for PRISMA flowchart).

**Table 2:** Search results from all six databases

Database	Interface	Dates	Results
MEDLINE	Ovid	2000–May 2017	2985
Embase	Elsevier	2000–May 2017	4722
Web of Science Core Collection	Thomson Reuters	2000–May 2017	4320
ERIC	ProQuest	2000–May 2017	796
PAIS Index	ProQuest	2000–May 2017	251
ABI/INFORM Global	ProQuest	2000–May 2017	1121
Total			14,195
Duplicates removed			4291
De-duplicated total (total screened at title/abstract level)			9904

Articles were excluded during full-text screening if they did not provide details on the actions contributing to their ES process, if the partnership was not between researchers and public policy decision-makers, and if the partnership was not an explicit and enduring research collaboration aiming to inform public policy. For example, some studies were excluded as they were a project without a long-term collaboration or because they focused on participatory policy-making. A final 29 articles were initially included but 18 of these were excluded from further analysis as they were deemed to be structured as a ‘research network’ with a shared domain of interest and collective outcomes rather than an explicit ‘research partnership’ that was focused on carrying out specific project activities within a scope of agreed-upon outcomes. The characteristics of the final 11 studies are described in Table 3.

**Table 3:** Descriptive information on final included studies

Primary author, year; country	Public policy issue	Partnership model/methods to describe and evaluate	Stakeholders	Initiator	Funding/duration	Key actions to support engaged scholarship	Contextual factors	Outcomes reported
Bowes et al., 2004 [32]; Australia	Child care and early childhood development	Partnership; ‘user-centric’ description of partnership (no evaluation)	Research: Australian Research Council; Macquarie University Public Policy: NSW Department of Community Services; Office of Child Care (commissioned study) Other: Sydney Day Nursery Association Children’s Services, Kindergarten Union Children’s Services; Practitioners	Request from Office of Child Care in the NSW Department of Community Services	NSW Department of Community Services contributed financial and in-kind support / more than 3 years	Meetings, funding for travel to annual meetings, six-monthly newsletter, teleconferences, expert policy personnel; all members planned stages of data collection and analysis	NR	NR; calls for future research applying an ecological approach with many contextual factors
Bumbarger et al., 2012 [33]; United States	Children’s mental health	Research–policy partnership description of partnership (no evaluation)	Research: Prevention Research Center at Penn State University Public Policy: Pennsylvania Commission on Crime and Delinquency, Department of Public Welfare, State Departments of Education and Health, Juvenile Court Judges Commission	Penn State Prevention Research Center	Pennsylvania Commission on Crime and Delinquency, Department of Public Welfare, State Departments of Education and Health / Decade long	Training of stakeholders for data analysis, stakeholder programme training	Effective communication; understanding and recognition of each partner’s hierarchy in project planning	Provided practical knowledge on partnership
Eriksson et al., 2014 [34]; Sweden	Health promotion	Academic practice policy partnership analysis of three case studies; realist approach including data from reflective dialogues, evaluation meetings and interviews	Research: Team at Orebro University Public Policy: National Board of Health and Welfare, followed by the National Institute of Public Health and, from 2014, by the Public Health Agency of Sweden	Researchers	NR / 2003 onwards	Consultations, conferences, project leader meetings each year; implementation of annual work programmes, annual progress reports on non-government organisation projects and research	Outlining responsibilities at beginning; limited previous experience working with non-government organisations	Final results presented and discussed with non-government organisations; in-depth studies, including two doctoral dissertations and published papers
			Research: Team at Orebro University: Public Policy: Sweden’s National Institute of Public Health; the Swedish Association of local Authorities and regions, and the Swedish Association of Municipal Housing Companies Other: The Swedish Research Council for Environment, Agricultural Sciences and Spatial Planning	Partnership agreement and a research programme were developed at the same time	National Institute of Public Health and small payment by each of the partners / 2003–2009	A steering group (politicians, public health officers, researchers), a coordinating committee (public health officers, researchers), working groups, and annual conferences	Policy and research stakeholders had previously been involved with a national network for public health action in larger municipalities in Sweden; common interests and perspectives	One doctoral dissertation and research papers
			Research: Team at Orebro University Public Policy: National Institute for Public Health; politicians within a municipality Other: practitioners at the public health administration in Karlskoga and Degerfors	The National Institute of Public Health issued call for research by municipality in collaboration with an academic institution.	NR / NR	Steering group and joint working group (academics, practitioners and politicians in the municipalities) that met monthly	Trust of researchers indicated by previous records; relevance and quality of research achievements	Nine research studies and two meta-analytic studies, a family guide and a book in Swedish, and presentations at national and international conferences
Jose et al., 2017 [35]; Australia	Workplace health promotion and policy decision-making	Partnership case study design and mixed-methods approach using partnership assessment tools and interviews	Research: University of Tasmania Public Policy: Tasmanian State Service	Tasmanian state government allocated funding, the National Health and Medical Research Council provided grant to evaluate and improve research partnership	Australia’s Partnership for Better Health Grants scheme/5 years	Quarterly meetings of management committee (researchers and public policy); broader investigator group; four working groups in areas of need; use of a partnership analysis tool; joint planning sessions to clarify research priorities and ensure research was policy relevant	Recognition of different research and policy priorities; flexibility and acknowledgment of different perspectives	Individualised reports for departments; 7 published papers; over 17 presentations at national and international conferences; presentations at local forums; lunchtime seminar series presented by researcher and policy-maker/manager
Bates et al., 2008 [36]; Canada	Telehealth solutions for cardiovascular disease	Alliance, partnership description of partnership (no evaluation)	Research: 15 university-based researchers from 4 universities Public policy: health authority policy-makers; the Vancouver Coastal Health Authority, The Northern Health Authority, The Provincial Health Services Authority Other: healthcare professionals	From a core group of researchers that identified the need for new models of care	Seed grant; external research funding sources / more than 2 years	Team leader and governance structure with responsibilities for admin and operational activities of partnership; communication plan of bimonthly teleconferences and quarterly face-to-face meetings	Maintaining communication; team leader facilitated involvement of patient front-line provider, collaboration and connections between stakeholders	Publication; innovation fund awarded for further development
Maluka et al., 2014; Tanzania [37]	Health systems and policy-making	Action research description of partnership (no evaluation)	Research: Tanzanian institutes, research institutions from Europe Public Policy: Council Health Management Team; Council Health Service Board Other: Involvement of groups from community district health setting, non-government organisations, community members	Researchers in Tanzania and Europe teamed with decision-makers	European Union/5 years	Priority-setting meetings; annual workshops; monthly reports; full-time person to facilitate the implementation of the project	Action research methodology required more meetings to guide council health management team	NR
Newman et al., 2011 [38]; Australia	Social exclusion	Network, research–policy collaboration description of partnership (no evaluation)	Research: Flinders University Public policy: policy actor from social inclusion unit located within government in the Department of the Premier and the cabinet	WHO established the Commission on Social Determinants of Health, setting up nine knowledge networks – The Social Exclusion Knowledge Network	NR / 3 months	Weekly visits to government offices allowing for discussion with key policy actors; face-to-face discussions and side-by-side work on joint work; post project de-briefing meetings; key researcher liaison responsible for timely completion and consultation with partners	Contextual elements of researcher–policy partnership included developing relationship (working together early); acknowledging and appreciating cultural differences; clarifying the goal; defining the roles; creating the process and the knowledge together; deriving implications from the knowledge	Final report was produced
Rütten et al., 2014 [39]; Germany	Physical activity promotion project	Capacity-building interactive knowledge-to-action case study - process evaluation (participant observation, interviews, survey)	Research: University-based research Public policy: Ministry of Health; regional-level public-law institutions Other: non-government organisation	Scientific partner teamed up with relevant organisations	European Commission/NR	Co-operative planning; team building through teaming research and policy partners; planning group-involved sessions to brainstorm, prioritise ideas, define goals, develop specific actions to reach goals	Scheduling challenges - smaller organisations had limited staff and larger organisations sent different representatives, which hampered the continuity of the process	NR
Theobald et al., 2009 [40]; Africa	Case #1: HIV counselling and testing in Kenya	Research–policy/practice interface, OPERATIONAL research case study – used RAPID framework to analyse factors influencing research into policy	Research: Liverpool School of Tropical Medicine’s Global Health Development Group Public policy: Government of Kenya	Emerged from Ministerial Summit of Health Research in Mexico City in 2004	NR / NR	Capacity-building activities to consolidate linkages and partnership; teaching and supervision systems facilitated constructive engagement with programme planners; established national taskforce; involved counsellors in testing of guidelines; incorporated clients’ concerns into guidelines	Data availability; capacity-building	Noted lack of funding to implement recommendations provided from the research
	Case #2: provision of tuberculosis services in grocery stores in Malawi		Research: Liverpool School of Tropical Medicine’s Global Health Development Group Public policy: Ministry of Health Malawi: policy-makers Other: Research for Equity and Community Health		Norwegian Heart and Patient Lung Association / NR			
	Case #3: community diagnosis for anaemia, tuberculosis and malaria in Nigeria		Research: Liverpool School of Tropical Medicine’s Global Health Development Group Public policy: Federal and State Health Ministries: policy-makers		Department for International Development / NR			
Tran et al., 2009 [41]; Malaysia	Road traffic injuries	Collective research and practice, collaborative learning framework description of partnership (no evaluation)	Research: Universiti Putra Malaysia in Malaysia, and the Johns Hopkins University Bloomberg School of Public Health in the United States Public policy: Ministry of Transport, Malaysian Institute for Road Safety Research	Researchers held meetings with Department of Road Safety and other stakeholders	FIA Foundation, Malaysian Department of Road Safety, the Selangor State Road Safety Council, the Klang District Municipality	Knowledge brokering, included (1) synthesis of available research; (2) policy analysis involving various stakeholders, range of policy options developed; (3) policy/research forum to determine policy recommendations; followed framework for collective research practice; plan for dissemination of the results developed to inform stakeholders; national dissemination workshop; public meetings to identify vision, goal and objectives	Guided by a framework; Government endorsement; differences in problem solving approaches and perspectives	Strengthened relationships and opened up communication between academic researchers and policy-makers to support future collective research and practice
Waqa et al., 2013 [42]; Fiji	Obesity prevention in communities	Knowledge brokering case study - data collected through process diaries describing interaction	Research: Fiji National University; Fiji School of Medicine in Suva; Deakin University Public Policy: four government departments Other: two non-government organisations	Project managed by researchers at Fiji National University, the Pacific Research Centre for the Prevention of Obesity and Non-communicable Diseases and Deakin University	Australian Agency for International Development on an Australian Development Research Awards grant / 2009–2012	Emails, telephone conversations; nomination of advisors to facilitate activities; workshops; part-time research fellow and consultant hired to assist with workshops and provide support to advisory groups	Some organisations had limited access to online databases	Development of evidence-informed policy briefs aligned with national and organisational strategies; oral presentations of the brief and submission of written document to high-level officers/decision-makers

*NR* none reported, *NSW* New South Wales

The included studies were from research partnerships in various countries, including Australia (*n* = 3), Canada (*n* = 1), Fiji (*n* = 1), Germany (*n* = 1), Malaysia (*n* = 1), Sweden (*n* = 1), Tanzania (*n* = 1) and the United States (*n* = 1). Another study included three case studies from African countries, including Kenya, Malawi and Nigeria. None of the included studies explicitly used the term ‘engaged scholarship’ but were deemed to fit the inclusion criteria for an ES partnership. Various terms were used to describe the partnerships, including action research, alliance, research or academic policy/practice and collective research. Various public policy partners were engaged in research, including those from national, state/provincial and local governments or authorities. Seven studies also engaged other stakeholders such as those from non-government or community organisations, research councils and practitioners. Six included studies were descriptive case studies, without evaluation methods used to systematically collect information on the partnership process or outcomes. Four studies used process evaluation methods to collect information on the partnership, including participant observations, surveys, process diaries, reflective dialogues and meeting notes. One study used a framework to analyse the characteristics of case studies using a framework for research-policy linkages.

Various actions and contextual factors were identified in the included studies as contributing to ES partnerships. With respect to actions, the importance of frequent interactions with public policy stakeholders and co-planning and executing research was discussed as being important in all partnerships. Common methods for interactions included regular email communications, in-person meetings or teleconferences. One study also described weekly visits to government offices which allowed for ongoing and face-to-face discussion with policy actors [38]. Several partnerships held capacity-building for stakeholders such as training for data analysis [33], conferences with stakeholders [34] and a national dissemination workshop to share findings of the research [41]. Steering groups, coordinating or management committees and working groups were discussed in several studies as facilitating partnership development [34–36]. Bates et al. [36] further developed a governance structure with members responsible for administrative and operational activities of partnerships and a communication plan. Several studies also discussed appointing team leaders/champions [34, 36–38, 42] or expert/technical personnel from stakeholder groups [32, 36] to support partnered research. Communication planning and priority-setting were other reported actions within the included studies [37, 39].

Clarity in responsibilities and respect for partner priorities were key contextual factors that influenced the partnerships in the included studies [33–36, 38]. One study discussed the lack of clarity of their action research methodology, which required more meetings to guide the management team [37]. Tran et al. [41] commented on the differences in perspectives that may create challenges for collaboration, whereas other studies discussed the importance of flexibility in partnerships to allow for acknowledgement and appreciation of cultural differences among stakeholders [35, 38]. Prior experience or relationships among stakeholders and credibility of the researcher were reported to also facilitate partnership development [34, 38]. Contextual limitations related to timing or readiness of stakeholders and continuity of policy actors was also discussed in one study [39].

## Discussion

This study used a scoping review methodology to articulate the actions and context that support the coproduction and use of research knowledge to inform public policy decisions. The purpose was to identify strategies for researchers and policy stakeholders working in ES partnerships to enhance their collaborative research efforts and ascertain key areas that require future investigation. We intentionally used the term ‘ES’ to connect it with an engagement paradigm, compared to a knowledge transfer paradigm, with its biomedical roots and tendency to focus on communication and dissemination by researchers [9]. In contrast, ES focuses on collaboration and integrating diverse perspectives to create more relevant and usable research [1]. Notwithstanding this intent, none of the included studies defined their partnership as ES. However, they all met the inclusion criterion that operationalised ES, namely a partnership where researchers were working with individuals working in public policy toward the same end goal of coproduction and use of knowledge.

Key actions included facilitating frequent interactions with public policy stakeholders and joint planning and execution of data collection and analysis. Critical contextual factors influencing partnerships were clarity in responsibilities, prior relationships or experiences, and respect for partner priorities and perspectives. The application of an ES approach is expected to result in more relevant research through coproduction between researchers and policy actors, thereby increasing the likelihood that the evidence will be used to inform policy decisions. Although the included studies discussed how the results would lead toward public policy decisions, the outcomes reported in the included studies focused on academic outputs, including research papers, dissertations and conference presentations with some additional policy-relevant products such as presentation at local forums, individualised reports or local discussions with stakeholders. None of the included studies specifically articulated how the research led to a policy change; however, this could be due to the nature of timing of the publication and that policy outcomes from ES may take more time to achieve.

The partnership, actions and context were often incomplete and inconsistency was reported. In particular, we were interested in understanding the ‘embeddedness’ of the researcher in the policy environment. ‘Embedded research’ is one action that may facilitate ES as it situates a researcher within a policy setting to conduct evaluation and research as a member of the host organisation [43, 44]. This enables the researcher to be independent from the host organisation but familiar with the policy context, providing an opportunity to engage in critical analysis and bridge knowledge-to-action gaps. For example, Newman et al. [38] described weekly visits to government offices allowing for face-to-face discussion with key policy actors and joint work. The study referred to key elements critical to the success of researcher and public policy partnerships, including developing the relationship and creating the process and knowledge together. These findings reflect the potential importance of greater embeddedness of research within policy, although the other studies in the review did not provide sufficient detail on the context of the partnership to determine the extent to which researchers were embedded in the policy environment. Further, half of the included studies did not use an evaluation method in their study (including Newman et al. [38]), meaning that the actions and context were not systematically collected and may be biased through the accounts of the authors. As a result of these limitations in the literature, it is not possible to determine the relationships between actions, context and outcomes for ES. This is a similar finding by Gagliardi et al. [14] who advocated for more longitudinal approaches to determine the impact of partnerships in research and relationships between approaches and outcomes. The results from this scoping review suggest that further study is needed to more systematically understand the relationships between contexts and actions. It is also recommended that future studies articulate the nature of ES partnerships, including the embeddedness of researchers within policy environments, and use evaluative methods to collect perspectives from the various stakeholders involved to reduce the bias of researcher accounts.

This study is a novel contribution to the literature by focusing on partnerships between researchers and public policy decision-makers on a variety of topics. Previous reviews have explored healthcare research partnerships [14, 45, 46] and community-based participatory research [8]. The scoping review identified public policy issues across multiple sectors, including early childhood, mental health, health promotion, healthcare, social exclusion and safety. This transdisciplinary approach to the review provides an opportunity to learn about partnership approaches across sectors. An additional 18 studies were excluded due to their structure as a ‘research network’. This boundary was necessary to keep the review focused on partnerships that carried out specific project activities with agreed-upon outcomes rather than broad collectives that engage researchers and public policy stakeholders with shared domains of interest but without tangible activities. However, the research network studies were charted in the preliminary stages of the review and similar actions and contexts were reported. These studies also represented multiple sectors, such as health policy, healthy aging and long-term care, public education, regional water issues, wildlife conservation and family violence. One noted difference in the ‘research network’ studies included further actions related to stakeholder capacity and relationship building, such as workshops and large assemblies. Additional contextual challenges were also mentioned that related to tension and conflicts working within a large research network and balancing the needs of different stakeholders.

## Conclusions

In conclusion, more systematic study of the conditions and actions that influence the coproduction and use of research is needed for a diversity of public policy issues. ES partnerships could be further explored beyond the focus on public policy issues to determine the use of this partnership approach within practice-oriented literature. Future research could build on the inherent limitations of the scoping review methodology given their exploratory nature and uncertainty about interpretation due to the lack of quality appraisal [47]. More comprehensive descriptions (including the duration) and evaluations of research partnerships are also needed to elucidate the contextual and process factors that contribute to the research–policy partnerships and to determine the long-term outcomes of its use, given the limited available evidence [24, 48, 49]. This advancement in the literature would help to strengthen research–policy partnerships and their intended outcomes.

## Supplementary information

**Additional file 1. **Search translation summary.

**Additional file 2. **PRISMA 2009 flow diagram.

## Data Availability

Not applicable.

## Abbreviations

ES: Engaged scholarship
