# Supplementary material for: Engaged scholarship and public policy decision-making: a scoping review
Source: Health Res Policy Syst. 2020 Aug 26;18:96. doi: 10.1186/s12961-020-00613-w (PMC7449077; doi:10.1186/s12961-020-00613-w)
Supplement: Supplementary file 1 — Additional file 1. Search translation summary. [file 12961_2020_613_MOESM1_ESM.docx]

**Additional file 1 – Search Translation Summary**

# MEDLINE (Ovid)

### Search strategy:

|  | **Searches** |
| --- | --- |
| 1 | Decision Making/ |
| 2 | Decision Making, Organizational/ |
| 3 | government.tw. |
| 4 | exp Government/ |
| 5 | (policy-mak* or policymak*).tw. |
| 6 | (decision-mak* or decisionmak*).tw. |
| 7 | ((decision? or policy or policies) adj2 (make or maker? or making)).tw. |
| 8 | exp Policy Making/ |
| 9 | or/1-8 |
| 10 | Cooperative Behavior/ |
| 11 | Interinstitutional Relations/ |
| 12 | Interprofessional Relations/ |
| 13 | Partner?.tw. |
| 14 | Partnership?.tw. |
| 15 | Collaborat$3.tw. |
| 16 | cooperat$3.tw. |
| 17 | or/10-16 |
| 18 | Research Personnel/ |
| 19 | exp Evidence-Based Practice/ |
| 20 | Researcher?.tw. |
| 21 | or/18-20 |
| 22 | 9 and 17 and 21 |

# Embase (Elsevier)

### Search strategy:

('decision making'/de OR government:ab,ti OR 'government'/exp OR ('policy mak*':ab,ti OR policymak*:ab,ti) OR ('decision mak*':ab,ti OR decisionmak*:ab,ti) OR ((decision? OR policy OR policies) NEAR/2 (make OR maker? OR making)):ab,ti OR 'policy'/exp) AND ('cooperation'/exp OR 'public relations'/exp OR 'public-private partnership'/exp OR (partner:ab,ti OR partners:ab,ti) OR (partnership:ab,ti OR partnerships:ab,ti) OR collaborat*:ab,ti OR cooperat*:ab,ti) AND ('scientist'/exp OR 'evidence based practice'/exp OR (researcher:ab,ti OR researchers:ab,ti))

# Web of Science (Core Collection) (Thomson Reuters)

### Search Strategy:

| # 16 | **3,796** | #5 AND #11 AND #15 |
| --- | --- | --- |
| # 15 | 285,743 | #12 OR #13 OR #14 |
| # 14 | 225,754 | TS=(researcher$) |
| # 13 | 50,532 | TS=(evidence based practice) |
| # 12 | 13,261 | TS=(Research Personnel) |
| # 11 | 655,558 | #6 OR #7 OR #8 OR #9 OR #10 |
| # 10 | 266,663 | TS=(cooperat*) |
| # 9 | 226,974 | TS=(collaborat*) |
| # 8 | 53,633 | TS=(partnership$) |
| # 7 | 154,846 | TS=(partner$) |
| # 6 | 15,688 | TS=(cooperative behavio$r) |
| # 5 | 573,602 | #1 OR #2 OR #3 OR #4 |
| # 4 | 339,621 | TS=((decision$ or policy or policies) NEAR/2 (make or maker$ or making)) |
| # 3 | 248,163 | TS=(decision-mak* or decisionmak*) |
| # 2 | 59,453 | TS=(policy-mak* OR policymak*) |
| # 1 | 238,032 | TS=(government) |

# ERIC (ProQuest)

### Search strategy:

ti((SU.EXACT("Participative Decision Making") OR government OR SU.EXACT("Government (Administrative Body)") OR (policy-mak* OR policymak*) OR (decision-mak* OR decisionmak*) OR ((decision? or policy or policies) NEAR/2 (make or maker? or making)) OR SU.EXACT("Policy Formation")) AND (SU.EXACT("Cooperative Planning") OR SU.EXACT("Institutional Cooperation") OR SU.EXACT("Interprofessional Relationship") OR Partner? OR Partnership? OR Collaborat$3 OR cooperat$3 OR SU.EXACT("Theory Practice Relationship")) AND (Researcher? OR (SU.EXACT("Researchers") OR Researcher?) OR (evidence based practice))) OR ab((SU.EXACT("Participative Decision Making") OR government OR SU.EXACT("Government (Administrative Body)") OR (policy-mak* OR policymak*) OR (decision-mak* OR decisionmak*) OR ((decision? or policy or policies) NEAR/2 (make or maker? or making)) OR SU.EXACT("Policy Formation")) AND (SU.EXACT("Cooperative Planning") OR SU.EXACT("Institutional Cooperation") OR SU.EXACT("Interprofessional Relationship") OR Partner? OR Partnership? OR Collaborat$3 OR cooperat$3 OR SU.EXACT("Theory Practice Relationship")) AND (Researcher? OR (SU.EXACT("Researchers") OR Researcher?) OR (evidence based practice)))

# PAIS Index (ProQuest)

### Search Strategy:

(SU.EXACT("Decision Making" OR "Group Decision Making" OR "Participative Decision Making") OR government OR SU.EXACT("Government") OR (policy-mak* OR policymak*) OR (decision-mak* OR decisionmak*) OR ((decision? or policy or policies) NEAR/2 (make or maker? or making)) OR SU.EXACT("Policy Making")) AND (SU.EXACT("Cooperation") OR ((institution$2 OR interinstitution$2) NEAR/2 relation$4) OR ((professional OR interprofessional) NEAR/2 relation$4) OR SU.EXACT("Theory Practice Relationship") OR Partner? OR Partnership? OR Collaborat$3 OR cooperat$3) AND (SU.EXACT("Researchers") OR (evidence based practice) OR Researcher?)

# ABI/INFORM Global (ProQuest)

### Search strategy:

ti((SU.EXACT("Decision making") OR government OR SU.EXACT("Government") OR (policy-mak* OR policymak*) OR (decision-mak* OR decisionmak*) OR ((decision? or policy or policies) NEAR/2 (make or maker? or making)) OR SU.EXACT("Policy making")) AND (SU.EXACT("Cooperation") OR SU.EXACT("Interprofessional cooperation") OR Partner? OR Partnership? OR Collaborat$3 OR cooperat$3) AND (SU.EXACT("Researchers") OR (SU.EXACT("Evidence-based medicine") OR SU.EXACT("Evidence-based nursing")) OR (evidence based practice) OR Researcher?)) OR ab((SU.EXACT("Decision making") OR government OR SU.EXACT("Government") OR (policy-mak* OR policymak*) OR (decision-mak* OR decisionmak*) OR ((decision? or policy or policies) NEAR/2 (make or maker? or making)) OR SU.EXACT("Policy making")) AND (SU.EXACT("Cooperation") OR SU.EXACT("Interprofessional cooperation") OR Partner? OR Partnership? OR Collaborat$3 OR cooperat$3) AND (SU.EXACT("Researchers") OR (SU.EXACT("Evidence-based medicine") OR SU.EXACT("Evidence-based nursing")) OR (evidence based practice) OR Researcher?))
