# Supplementary material for: Engaged scholarship and public policy decision-making: a scoping review
Source: Health Res Policy Syst. 2020 Aug 26;18:96. doi: 10.1186/s12961-020-00613-w (PMC7449077; doi:10.1186/s12961-020-00613-w)
Supplement: Supplementary file 2 — Additional file 2. PRISMA 2009 flow diagram. [file 12961_2020_613_MOESM2_ESM.doc]

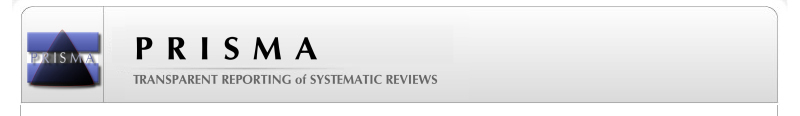
**PRISMA 2009 Flow Diagram**

**Screening**

**Included**

**Eligibility**

**Identification**

Records identified through database searching
(n = 14195)

Records after duplicates removed
(n = 9904)

Records screened
(n = 9904)

Records excluded
(n = 9529)

Full-text articles assessed for eligibility
(n = 375)

Full-text articles excluded (n = 346)

Studies identified as relevant (n = 29)

Final studies included in synthesis

(n = 11)

Articles excluded due to partnership focus as a network (n = 18)
